# Relationship between Oxidant/Antioxidant Markers and Severity of Microalbuminuria in the Early Stage of Nephropathy in Type 2 Diabetic Patients

**DOI:** 10.1155/2013/232404

**Published:** 2013-02-26

**Authors:** Ning Shao, Hong Yu Kuang, Na Wang, Xin Yuan Gao, Ming Hao, Wei Zou, Hui Qing Yin

**Affiliations:** ^1^Department of Endocrinology, The First Affiliated Hospital of Harbin Medical University, Harbin, Heilongjiang 150001, China; ^2^Department of Endocrinology, The Affiliated Hospital of Jining Medical University, Jining, Shandong 272000, China; ^3^Department of Neurology, The First Affiliated Hospital of Heilongjiang University of Chinese Medicine, Harbin, Heilongjiang 150040, China

## Abstract

A wide range of microalbuminuria cutoff values are currently used for diagnosing the early stage of nephropathy in type 2 diabetes (T2D). This study analyzed the relationships between oxidant and antioxidant markers of nephropathy and the severity of microalbuminuria. The study included 50 healthy controls (Group 1), 50 diabetic patients with no nephropathy (Group 2), 50 diabetic patients with nephropathy and a urinary albumin excretion (UAE) of 30–200 mg/24 h (Group 3), and 50 diabetic patients with UAE 200–300 mg/24 h (Group 4). Serum nitrotyrosine, conjugated dienes, 8-hydroxy-2′-deoxyguanosine (8-OHdG), superoxide dismutase (SOD), and total antioxidant capacity (T-AOC) levels were determined. Oxidative stress is increased in the early stage of nephropathy in patients with T2D. There was a significant correlation between the extent of microalbuminuria and markers of oxidative stress. Multiple linear regression analysis identified lipid oxidative stress as a possible independent marker for evaluating the degree of renal damage in diabetic nephropathy. Stratifying microalbuminuria values during the early stage of nephropathy might be an important factor in facilitating earlier and more specific interventions.

## 1. Introduction

Diabetic nephropathy (DN) is an important microvascular complication of diabetes and is widely recognized as the most common cause of the end-stage renal disease seen in clinical practice. The condition not only causes disability but is associated with a high mortality rate in diabetic patients as well [1]. Early treatment depends on a clear understanding of the mechanisms that underlie DN. Oxidative stress is becoming increasingly recognized as an important causative factor. Routine urine testing fails to detect DN in its early stages even though urinary protein results may be positive. This means that treatment is delayed and that therapeutic responses are far from optimal. 

 An imbalance between oxidation and antioxidation is thought to precede the development of renal lesions, and thereafter the degree of oxidation gradually increases in parallel with the progression of the disease [2]. Previous studies have shown that the extent of microalbuminuria affects long-term prognosis [3]. The emergence of microalbuminuria not only indicates the early stage of DN, but may also be the consequence of extensive damage of systemic endothelial cells [4]. These studies confirmed that although microalbuminuria was currently the best nontraumatic predictor of early stage of DN, advanced changes in renal structure may have already developed when microalbuminuria is first seen. It has also been shown that reducing urinary protein excretion is of therapeutic benefit to patients with clinically significant microalbuminuria [5]. However, the normal values for microalbuminuria currently used to diagnose DN may be too high, and the range of microalbuminuria values is probably too wide [6].

 These findings highlight the need to clarify the relationship between markers of oxidative stress and severity of microalbuminuria based on urinary albumin excretion (UAE). This information would help to define a specific target for the prevention and treatment of DN.

 In the present study, we determined the association between levels of oxidant and antioxidant markers and different degrees of microalbuminuria in patients with type 2 diabetes (T2D) and an early stage of nephropathy. We used multiple linear regression analysis to identify the predominant risk factors that determined the severity of microalbuminuria.

## 2. Materials and Methods

### 2.1. Subjects and Groups

Between December 2007 and December 2010, 200 subjects 18 years of age or older were recruited into the study from the Health Examination Center and the Department of Endocrinology at The First Clinical Hospital of Harbin Medical University. The study subjects were divided into four groups. Group 1 comprised 50 healthy control subjects without diabetes, Group 2 included 50 patients with T2D who did not have nephropathy, Group 3 comprised 50 patients with T2D and nephropathy with an UAE between 30 and 200 mg/24 h, and Group 4 included 50 patients with T2D and an UAE between 200 and 300 mg/24 h. Patients and control subjects were well generally matched in terms of demographic and physiological parameters (Table 1).

 The diagnosis of type 2 diabetes mellitus was based on the 1999 World Health Organization (WHO) diagnostic criteria [7], and the presence of early-stage DN was based on the staging criteria proposed by Mogensen et al. [8]. The diagnostic criterion for microalbuminuria was an UAE between 30 and 300 mg/24 h, measured at least twice from overnight urine samples taken three times in the 6 months before entering the study. All control subjects adhered to a standardized diet based on 2007 China guideline for type 2 diabetes [9]. 

 Pregnant and lactating women and diabetic patients with ketoacidosis or inadequately controlled blood glucose were excluded from entering the study. None of the patients had changes in urinary protein levels >50% in the 2 weeks before entering the study and none had proteinuria caused by heart, liver, kidney, urinary tract infection, or other diseases. In addition, none of the patients had received antioxidant drugs (i.e., vitamin E), antihypertensive medication, or lipid-lowering medication for at least 1 month prior to entering the study. 

 The study was conducted in accordance with the ethical principles of Declaration of Helsinki and was approved by the Ethical Committee of The First Clinical Hospital of Harbin Medical University. All participants provided informed consent prior to entering the study.

### 2.2. Assessments

Blood samples were obtained after an overnight fast. Samples (3 mL) of venous blood were incubated in a water bath for 30 minutes at 37°C and centrifuged at 4500 rpm for 10 minutes. The supernatants were stored at −70°C. Serum nitrotyrosine, conjugated dienes, and 8-hydroxy-2′-deoxyguanosine (8-OHdG) levels were measured by enzyme-linked immunosorbent assay (ELISA), in accordance with the procedures described in the assay kit (Netherlands HyCult Biotechnology b.v (Hbt). Serum superoxide dismutase (SOD) activity and total antioxidant capacity (T-AOC) levels were determined by spectrophotometry (TU-1900 UV-visible spectrophotometer). The assays were conducted according to the manufacturer's (Nanjing Blanching Institute of Biology) instructions. 

 Triglyceride, total cholesterol, high-density lipoprotein cholesterol (HDL-C), low-density lipoprotein cholesterol (LDL-C), fasting plasma glucose, and glycosylated hemoglobin (HbA1c) levels were measured using routine clinical chemical assays. 

### 2.3. Statistical Analysis

The statistical analysis was undertaken using SPSS version 17 software. The data were presented as means ± standard deviations (SDs). The variables were tested for normality using the Kolmogorov-Smirnov test. Parametric variables were compared using one-way analysis of variance (ANOVA). Associations between degree of microalbuminuria and measured parameters were analyzed using the Pearson correlation test. Multiple linear regression analysis was undertaken to identify independent risk factors. Values of *P* < .05 were considered statistically significant.

## 3. Results

Fasting plasma glucose levels and HbA1c levels were well matched between the three groups of diabetic patients and microalbuminuria levels (mg/24 h) reflected the levels intended in the study design (Table 1). In addition, patients in Group 2 had comparable levels of microalbuminuria as healthy control subjects. 

 As shown in Table 2, serum nitrotyrosine, conjugated dienes, and 8-OHdG levels were significantly higher in Groups 2, 3, and 4 than in Group 1 (*P* < .001), whereas SOD and T-AOC levels were significantly lower in Groups 2, 3, and 4 than in Group 1 (*P* < .001). Serum nitrotyrosine, conjugated dienes, and 8-OHdG levels were significantly higher and SOD and T-AOC were significantly lower in Group 3 than in Group 2 (*P* < .001). Levels of serum nitrotyrosine, conjugated dienes, and 8-OHdG were significantly higher in Group 4 than in Group 3 and SOD and T-AOC levels were significantly lower in Group 4 than in Group 3 (*P* < .001).

**Figure 1:**
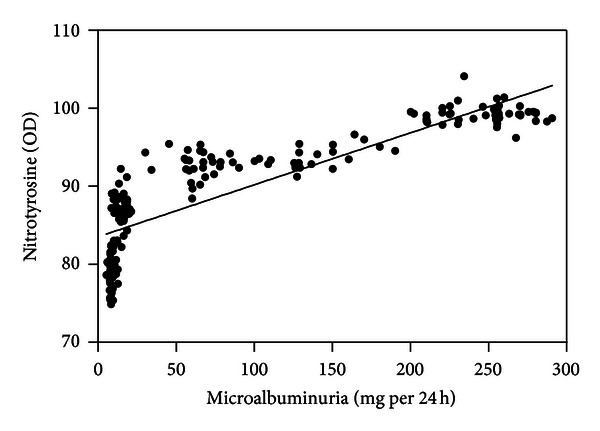
Correlation between microalbuminuria levels and nitrotyrosine.

**Figure 2:**
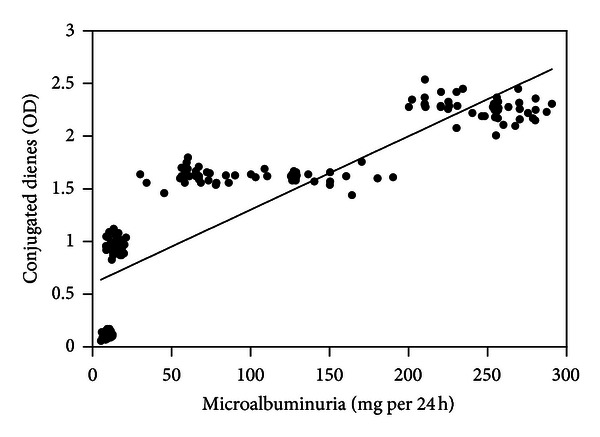
Correlation between microalbuminuria levels and conjugated dienes.

**Figure 3:**
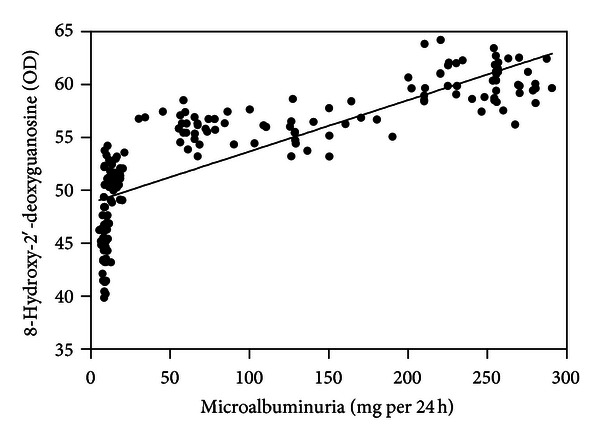
Correlation between microalbuminuria levels and 8-hydroxy-2′-deoxyguanosine.

**Figure 4:**
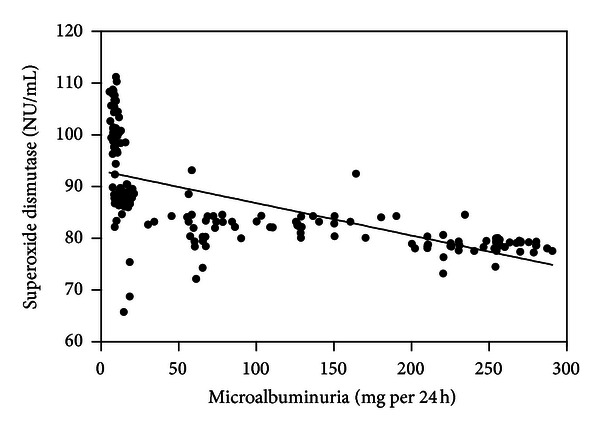
Correlation between microalbuminuria levels and superoxide dismutase.

**Figure 5:**
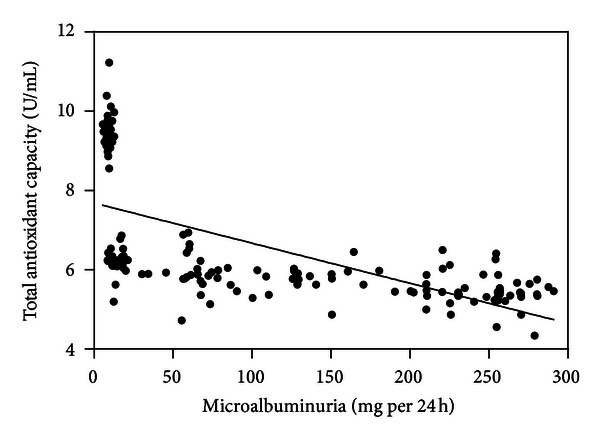
Correlation between microalbuminuria levels and total antioxidant capacity.

 Serum nitrotyrosine (*r* = 0.859; Figure 1), conjugated dienes (*r* = 0.867; Figure 2), and 8-OHdG levels (*r* = 0.826; Figure 3) were positively correlated with microalbuminuria (all *P* < .001). As shown in Figures 4 and 5, serum SOD (*r* = −0.659) and T-AOC levels (*r* = −0.609) were negatively correlated with microalbuminuria (*P* < .001). 

 Multiple linear regression analysis of microalbuminuria and oxidant and antioxidant markers identified conjugated dienes as the predominant indicator of the severity of microalbuminuria (Table 3). 

## 4. Discussion

We investigated the relationship between oxidative stress and severity of microalbuminuria in the early stages of nephropathy in patients with T2D. We were interested in finding out if different levels of microalbuminuria had clinical significance in patients in the same early stage of nephropathy. We also wanted to identify the main risk factor associated with the development of severe microalbuminuria. We, therefore, evaluated levels of different markers of oxidative stress in each of the four groups of subjects with different levels of microalbuminuria. We have selected different UAE cut-off values to analyze the relationship between oxidant/antioxidant markers and severity of microalbuminuria in an early stage of nephropathy in type 2 diabetic patients in our previous study, while UAE 30 mg/24 h and 200 mg/24 h were significantly for stratifying. There was a significant correlation between the extent of microalbuminuria and markers of oxidative stress when selecting UAE 30 mg/24 h; the results are consistent with UAE 200 mg/24 h. However, UAE 200 mg/24 h was significantly higher than UAE 30 mg/24 h; post hoc results the two values were selected as thresholds in the current study. 

 The generation of reactive oxygen species (ROS) is known to increase in parallel with the degree of oxidative stress and is responsible for oxidative damage to biological macromolecules (i.e., proteins, lipids, and nucleic acids) [10]. Oxidative damage to proteins is believed to play an essential role in the pathogenesis of many diseases [11, 12]. Nitrotyrosine is formed by the interaction of tyrosine residues with reactive nitrogen during the posttranslational modification of proteins. In pathological conditions, nitrotyrosine levels increase in response to the generation of reactive oxygen and reactive nitrogen species. Indeed, the formation of nitrotyrosine is considered as a biomarker of reactive nitrogen species in vivo. A study examining renal biopsy specimens demonstrated increasing levels of nitrotyrosine patients with DN, indicating that nitrotyrosine may be involved in the development of renal lesions in these patients [13]. 

 Conjugated dienes are lipid oxidation products, which can be further oxidized by polyunsaturated fatty acids found in low-density lipoprotein (LDL). Conjugated dienes' levels, therefore, indicate the degree of lipid oxidation. A number of studies have shown that serum conjugated diene levels are increased in patients with diabetes [14–16]. 

 Hydroxyl radicals and superoxide anions have been shown to interact with DNA molecules, resulting in breakage of the DNA chain, modification of DNA bases, and cross-linking of DNA proteins. These changes ultimately result in oxidative damage and produce 8-OHdG. Thus, 8-OHdG has been widely used as a marker for oxidative DNA damage. 

 Oxidative DNA damage plays an important role in the pathogenesis of many diseases (i.e., tumor, coronary artery disease, and diabetes) and has been shown to damage renal cells and mitochondrial DNA [17]. These changes may play a role in the development of DN. It has also been demonstrated that 8-OHdG levels are higher in diabetic patients than in healthy controls [16].

 Our study indicated that the serum levels of nitrotyrosine, conjugated dienes, and 8-OHdG were elevated in patients with early DN and diabetes without nephropathy and that the levels of these markers increased in parallel with the severity of microalbuminuria. These findings indicate that protein, lipid, and DNA damage already exist in the early stages of DN in patients with T2D and that the levels of oxidative stress increases as microalbuminuria becomes more pronounced. 

 The multiple linear regression analysis identified conjugated dienes as an oxidant marker of the risk of severe microalbuminuria. This finding suggests that the oxidative damage of lipids has the greatest impact and plays a fundamental role in the pathogenesis and progression of microalbuminuria in an early stage of DN. Although there were no significant differences in the lipid profile among the groups, we can speculate that visceral fat accumulation plays an important role in renal damage. Interestingly, a new study in China has shown that the expansion of visceral adiposity is a risk factor for an elevated risk of 24-hour UAE, and with the expansion of visceral adiposity, the prevalence of heavy albuminuria increases [18]. The results are consistent with a previous study demonstrating that the visceral fat area is independently associated with microalbuminuria in Japanese adult patients with T2D [19]. Abdominal obesity is an important feature of T2D patients in Asian populations, which is often accompanied by large amounts of visceral fat accumulation. It is those amounts of visceral fat accumulation that would cause lipid oxidative stress and promote adipose tissue to secrete inflammatory adipokines, such as interleukin- (IL)-6, tumor necrosis factor-*α* (TNF-*α*), and macrophage chemoattractant protein-1 (MCP-1) in an early stage of DN [20, 21]. 

 A decrease in antioxidant capacity breaks down the dynamic balance between oxidation and antioxidation in vivo. A growing amount of evidence indicates that the antioxidant capacity is decreased in diabetic patients [22, 23]. There are many enzymatic and nonenzymatic antioxidants in vivo, such as SOD, catalase (CAT), glutathione peroxidase, and vitamin E. Among these, SOD is the only substrate used as the superoxide anion scavenger enzyme, and as such it constitutes the first line of defense against ROS. T-AOC is an important marker of oxidation, which mainly reflects nonenzymatic but includes the activity of a minority of small molecular enzymatic systems. Animal and clinical studies have confirmed that antioxidant treatment plays an effective role in diabetes and DN [24–27]. Our results demonstrated that serum levels of SOD and T-AOC were lower than control subjects and decreased with severity of microalbuminuria in early DN and diabetes without nephropathy. These findings show that a decreased antioxidant activity already exists in diabetes at an early stage of DN and that antioxidant capacity weakens in parallel with the severity of microalbuminuria in T2D patients. 

 In conclusion, the oxidative stress increases in the early stage of nephropathy in patients with T2D relative to that in patients without nephropathy. However, in the same early stage of DN, there was a significant correlation between different levels of microalbuminuria and markers of oxidative stress. Serum conjugated dienes emerged as the main marker for evaluating kidney damage in DN. Stratifying microalbuminuria values during the early stage of nephropathy might be an important factor in facilitating earlier and more specific interventions. 

 The oxidative products assayed in our study are highly reactive, extremely unstable, and have a short half-life, all of which compromise accuracy. Our findings, therefore, need to be substantiated by other studies in larger numbers of patients from different centers.

## Figures and Tables

**Table 1:** Comparison of demographic and clinical characteristics (*n* = 50 per group).

Parameters	Group 1	Group 2	Group 3	Group 4
Age (years)	54.68 ± 6.47	55.66 ± 5.12	56.36 ± 3.97	56.98 ± 2.69
Female/male	27/23	28/22	25/25	26/24
Duration of diabetes (years)	—	7.3 ± 1.6	7.5 ± 1.8	7.7 ± 1.4
BMI (kg/m^2^)	24.24 ± 1.22	24.78 ± 1.50	24.54 ± 1.72	24.92 ± 1.66
Triglycerides (mmol/L)	1.46 ± 0.39	1.41 ± 0.11	1.38 ± 0.15	1.43 ± 0.10
Cholesterol (mmol/L)	4.48 ± 0.13	4.49 ± 0.48	4.58 ± 0.48	4.53 ± 0.55
HDL-C (mmol/L)	1.31 ± 0.14	1.29 ± 0.19	1.25 ± 0.20	1.26 ± 0.21
LDL-C (mmol/L)	1.39 ± 0.63	1.47 ± 0.87	1.55 ± 0.93	1.67 ± 1.01
Systolic blood pressure (mmHg)	122.92 ± 9.16	124.08 ± 6.87	123.36 ± 9.81	124.52 ± 5.61
Diastolic blood pressure (mmHg)	76.90 ± 4.13	77.10 ± 5.13	77.72 ± 5.64	78.04 ± 4.62
Microalbuminuria (mg/24 h)	8.82 ± 1.49	14.83 ± 3.27	96.73 ± 41.56^∗,†^	247.67 ± 24.01^∗,†,#^
Fasting plasma glucose (mmol/L)	4.96 ± 0.63	6.34 ± 0.74*	6.37 ± 0.82*	6.39 ± 0.76*
HbA1c (%)	5.93 ± 0.74	7.11 ± 0.50*	7.12 ± 0.63*	7.12 ± 0.61*

Mean ± standard deviation (SD), *P < .001 versus Group 1, ^†^ *P* < .001 versus Group 2, and ^#^ *P* < .001 versus Group 3.

**Table 2:** Comparison of markers of oxidant and antioxidant (*n* = 50 per group).

Parameters	Group 1	Group 2	Group 3	Group 4
Nitrotyrosine (OD)	79.01 ± 2.04	87.12 ± 1.89*	93.18 ± 1.16^∗†^	99.31 ± 1.15^∗†#^
Conjugated dienes (OD)	0.11 ± 0.02	0.97 ± 0.05*	1.62 ± 0.06^∗†^	2.27 ± 0.09^∗ †#^
8-OHdG (OD)	45.37 ± 2.52	51.45 ± 1.10*	55.94 ± 1.35^∗†^	60.40 ± 1.74^∗†#^
SOD (NU/mL)	101.27 ± 5.02	86.71 ± 4.89*	82.59 ± 3.37^∗†^	78.65 ± 1.52^∗†#^
T-AOC (U/mL)	9.50 ± 0.39	6.21 ± 0.23*	5.84 ± 0.41^∗†^	5.44 ± 0.39^∗†#^

Mean ± standard deviation (SD), *P < .001 versus Group 1, ^†^ *P* < .001 versus Group 2, and ^#^ *P* < .001 versus Group 3.

**Table 3:** Multiple linear regression analysis between microalbuminuria and oxidant and antioxidant markers.

Variable	*B*	SEB	Beta	*T*	*P* value
Nitrotyrosine	5.999	1.488	0.465	4.032	.000*
Conjugated dienes	148.771	18.664	1.201	7.971	.000*
8-OHdG	2.486	1.548	0.146	1.605	.010*
SOD	−1.688	0.677	−0.160	−2.491	.014*
T-AOC	−34.886	3.815	−0.580	−9.145	.000*
Constant	−881.492	142.025	—	−6.207	.000*

*Significant values.
